# Genome sequences of seven *Mannheimia haemolytica* isolates from feedlots in Oklahoma and California

**DOI:** 10.1128/mra.00785-24

**Published:** 2024-09-27

**Authors:** Margaret Krueger, Rinosh Mani, Srinand Sreevatsan

**Affiliations:** 1 Department of Pathobiology and Diagnostic Investigation, College of Veterinary Medicine, Michigan State University, East Lansing, Michigan, USA; 2 Bacteriology/Mycology Division, Veterinary Diagnostic Laboratory, Michigan State University, East Lansing, Michigan, USA; The University of Arizona, Tucson, Arizona, USA

**Keywords:** BRDC, *Mannheimia haemolytica*, *Mannheimia*, bovine respiratory disease, genome

## Abstract

This report describes the genome sequences of seven *Mannheimia haemolytica* isolates recovered from feedlot cattle. These genome sequences will enhance our understanding of *Mannheimia haemolytica* evolution and antimicrobial resistance (AMR)-associated genes they carried over a two-decade span.

## ANNOUNCEMENT

*Mannheimia haemolytica* is a naturally occurring pathogen frequently found in the upper respiratory tract of bovine and ovine species (1, 2). Commonly, stress induces *Mannheimia haemolytica* to opportunistically infect the respiratory tract, leading to bovine respiratory disease (BRD) (3). *Mannheimia haemolytica* has been detected in up to 75% percent of feedlot cattle with fatal BRD, leading to over $3 billion economic loss for the U.S. cattle industry when prevention, treatment, reduced production, and animal death costs are considered (4). (5–8).

Six archived *Mannheimia haemolytica* glycerol stocks (16–14, 37–14, 93–13a, 36–17, 41–13, and 67–13) from Oklahoma State University (isolation year: 2001) and one isolate (376 A1 CA) from California (isolated during 1990s) isolated from lung lesions of animals presenting with respiratory disease complex were revived on brain and heart infusion (BHI) with 5% defibrinated sheep blood agar at 37°C and 5% CO2 for 24 hours. The colonies were grayish with beta hemolysis under the colony. A single colony was selected and plated under the same conditions for further experiments. Isolates were confirmed as *Mannheimia haemolytica* with matrix-assisted laser desorption/ionization time of flight (MALDI-TOF). A single colony was added to 400 µL BHI broth and then plated on BHI blood agar. The plates were incubated at 37°C and 5% CO_2_ for 48 hours, and then a pellet was collected with a loop and suspended in 400 µL of BHI broth. Genomic DNA (gDNA) was isolated using the Zymo Research Quick-DNA HMW MagBead Kit (catalog number D6060). The isolates’ DNA and RNA concentrations were checked using a Qubit 4 fluorometer, and the A260/A280 and A260/A230 were checked using Nanodrop 1 before library preparation. The gDNA was prepared for sequencing using the Nanopore Rapid Barcoding Kit 24 V14 (SQK-RBK114.24). The gDNA was sequenced using a MinION R10.4.1 (FLO-MIN114) flow cell (FAY11803) on the Oxford Nanopore Technologies’ MinION Mk1C device, and base calling was done using the Fast model (400 bps), using software versions MinKNOW 24.02.16, Bream 7.9.8, Configuration 5.9.18, Dorado 7.3.11, and MinKNOW Core 5.9.12. with a 72 hour sequencing time.

The draft genomes were assembled using default settings unless mentioned. After base-calling, the generated reads were quality-assessed with NanoStat v1.6.0 (9), *de novo-*assembled with FLYE v2.9.3-b1797 (10), and polished with Medaka v1.11.2 (https://github.com/nanoporetech/medaka). The *de novo-*assembled contigs were checked for contamination with Kraken 2 (11). The draft genomes were quality-checked using Quast version 5.2.0 (12) and annotated using Prokka version 1.14.6 at beginner settings (13). Raw read and genome statistics can be found in Table 1.

**TABLE 1:** *Mannheimia haemolytica* idenification, raw read details, and genome data

Isolate	MALDI-ToF score (analysis 1/2)^*a*^^,^^*g*^	% *Mannheimia haemolytica^b^^,^^h^*	FastQC reads^*c*^	Read length N50 (bp)^*c*^	Total bp sequenced^*c*^	Phred score^*c*^^,^^*h*^	SRA accession	Contigs^*d*^	Contig N50 value (bp)^*d*^	Contig GC content (%)^*d*^	Coverage (X)^*e*^	Total sequence length (bp)^*d*^	Coding DNA sequences^*f*^	tRNAs^*f*^	rRNAs^*f*^	Genbank accession
16–14	2.39/2.48	85.76	331,706	6,790	1,128,540,697	10.5	SRR29808759	1	2702648	40.96	386	2702648	3700	65	20	CP163379
37–14	2.45/2.34	85.33	837,183	6,958	2,967,279,345	10.5	SRR29808756	1	2702211	40.97	991	2702211	3906	65	20	CP163378
93–13a	2.3/2.29	77.73	630,959	6,188	2,193,849,289	10.5	SRR29808760	1	2702533	40.97	708	2702533	3816	65	20	CP163377
36–17	2.46/2.49	94.78	570,668	8,057	2,168,393,628	10.5	SRR29808757	1	2694176	40.96	802	2694176	3766	63	20	CP163375
41–13	2.42/2.49	82.27	465,141	9,418	2,174,688,418	10.6	SRR29808755	1	2702367	40.97	746	2702367	3818	65	20	CP163374
67–13	2.37/2.44	85.16	411,047	8,975	1,658,786,524	10.4	SRR29808754	2	2702447	40.96	565	2725893	3819	65	20	JBFOHT000000000
376A1CA	2.21/2.46	93.85	558,884	9,687	2,458,430,655	10.5	SRR29808758	1	2697578	40.96	876	2697578	3833	63	20	CP163376

^*a*^ MALDI-TOF scores of 0.00–1.69 signify no reliable identification,1.70–1.99 identify to the genus level, and 2.00–3.00 reliably identify the organism to the genus and species levels.

^*b*^ Statistics generated using Kraken.

^*c*^ Statistics generated using NanoStat.

^*d*^ Statistics generated using Quast.

^*e*^ Statistics generated using Flye.

^*f*^ Statistics generated using Prokka.

^*g*^ Bacterial Cultures.

^*h*^ Raw reads.

All isolates were A1 serotype, found with https://ivsmlst.sund.ku.dk/ (14). Four isolates (16–14, 93–13a, 41–13, and 67–13) were found to have AMR-associated gene tet (H) by NCBI’s AMRFinderPlus (software v3.12.8, database v2024-01.31.1 (15), while the other isolates did not have any AMR genes.

The NCBI BioProject accession for the raw reads is PRJNA1127668. Table 1 has the SRA and GenBank accession numbers for the raw reads and assembled draft genomes, respectively.

## References

[1] Girma S, Getachew L, Beyene A, Tegegne DT, Tesgera T, Debelo M, Debano J, Teshome D, Abdisa K, Wirtu A, Tekle M, Abera B, Tafess K, Dandecha M, Abayneh T, Getachew B, Tufa TB, Tolera TS. 2023. Identification of serotypes of Mannheimia haemolytica and Pasteurella multocida from pneumonic cases of sheep and goats and their antimicrobial sensitivity profiles in Borana and Arsi zones, Ethiopia. Sci Rep 13:9008. doi:10.1038/s41598-023-36026-2 37268660 PMC10237525

[2] Magwood SE, Barnum DA, Thomson RG. 1969. Nasal bacterial flora of calves in healthy and in pneumonia-prone herds. Can J Comp Med 33:237–243. 4243028 PMC1319436

[3] Gonzalez CT, Maheswaran SK. 1993. The role of induced virulence factors produced by Pasteurella haemolytica in the pathogenesis of bovine pneumonic pasteurellosis: review and hypotheses. Br Vet J 149:183–193. doi:10.1016/S0007-1935(05)80088-0 8485643

[4] Jeyaseelan S, Sreevatsan S, Maheswaran SK. 2002. Role of Mannheimia haemolytica leukotoxin in the pathogenesis of bovine pneumonic pasteurellosis. Anim Health Res Rev 3:69–82. doi:10.1079/ahrr200242 12665107

[5] Ames TR. 1997. Dairy calf pneumonia. The disease and its impact. Vet Clin North Am Food Anim Pract 13:379–391. doi:10.1016/S0749-0720(15)30303-0 9368984 PMC7135296

[6] Booker CW, Abutarbush SM, Morley PS, Jim GK, Pittman TJ, Schunicht OC, Perrett T, Wildman BK, Fenton RK, Guichon PT, Janzen ED. 2008. Microbiological and histopathological findings in cases of fatal bovine respiratory disease of feedlot cattle in Western Canada. Can Vet J 49:473–481. 18512458 PMC2359492

[7] Lillie LE. 1974. The bovine respiratory disease complex. Can Vet J 15:233–242. 4370742 PMC1696627

[8] Wikse SE. 1985. Feedlot cattle pneumonia. Vet Clin North Am Food Anim Pract 1:289–310. doi:10.1016/s0749-0720(15)31328-1 3907776

[9] De Coster W, D’Hert S, Schultz DT, Cruts M, Van Broeckhoven C. 2018. NanoPack: visualizing and processing long-read sequencing data. Bioinformatics 34:2666–2669. doi:10.1093/bioinformatics/bty149 29547981 PMC6061794

[10] Kolmogorov M, Yuan J, Lin Y, Pevzner PA. 2019. Assembly of long, error-prone reads using repeat graphs. Nat Biotechnol 37:540–546. doi:10.1038/s41587-019-0072-8 30936562

[11] Wood DE, Lu J, Langmead B. 2019. Improved metagenomic analysis with Kraken 2. Genome Biol 20:257. doi:10.1186/s13059-019-1891-0 31779668 PMC6883579

[12] Mikheenko A, Prjibelski A, Saveliev V, Antipov D, Gurevich A. 2018. Versatile genome assembly evaluation with QUAST-LG. Bioinformatics 34:i142–i150. doi:10.1093/bioinformatics/bty266 29949969 PMC6022658

[13] Seemann T. 2014. Prokka: rapid prokaryotic genome annotation. Bioinformatics 30:2068–2069. doi:10.1093/bioinformatics/btu153 24642063

[14] Christensen H, Bisgaard M, Menke T, Liman M, Timsit E, Foster G, Olsen JE. 2021. Prediction of Mannheimia haemolytica serotypes based on whole genomic sequences. Vet Microbiol 262:109232. doi:10.1016/j.vetmic.2021.109232 34509701

[15] Feldgarden M, Brover V, Gonzalez-Escalona N, Frye JG, Haendiges J, Haft DH, Hoffmann M, Pettengill JB, Prasad AB, Tillman GE, Tyson GH, Klimke W. 2021. AMRFinderPlus and the Reference Gene Catalog facilitate examination of the genomic links among antimicrobial resistance, stress response, and virulence. Sci Rep 11:12728. doi:10.1038/s41598-021-91456-0 34135355 PMC8208984

